# 2016 ISCB Innovator Award: Serafim Batzoglou

**DOI:** 10.1371/journal.pcbi.1004973

**Published:** 2016-07-07

**Authors:** Christiana N. Fogg, Diane E. Kovats

**Affiliations:** 1 Freelance Science Writer, Kensington, Maryland, United States of America; 2 Executive Director, International Society for Computational Biology, Bethesda, Maryland, United States of America

**Figure:**
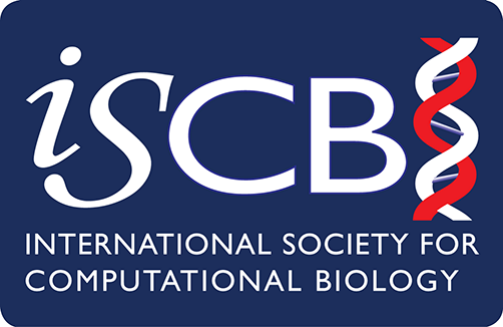


**Fig 1:**
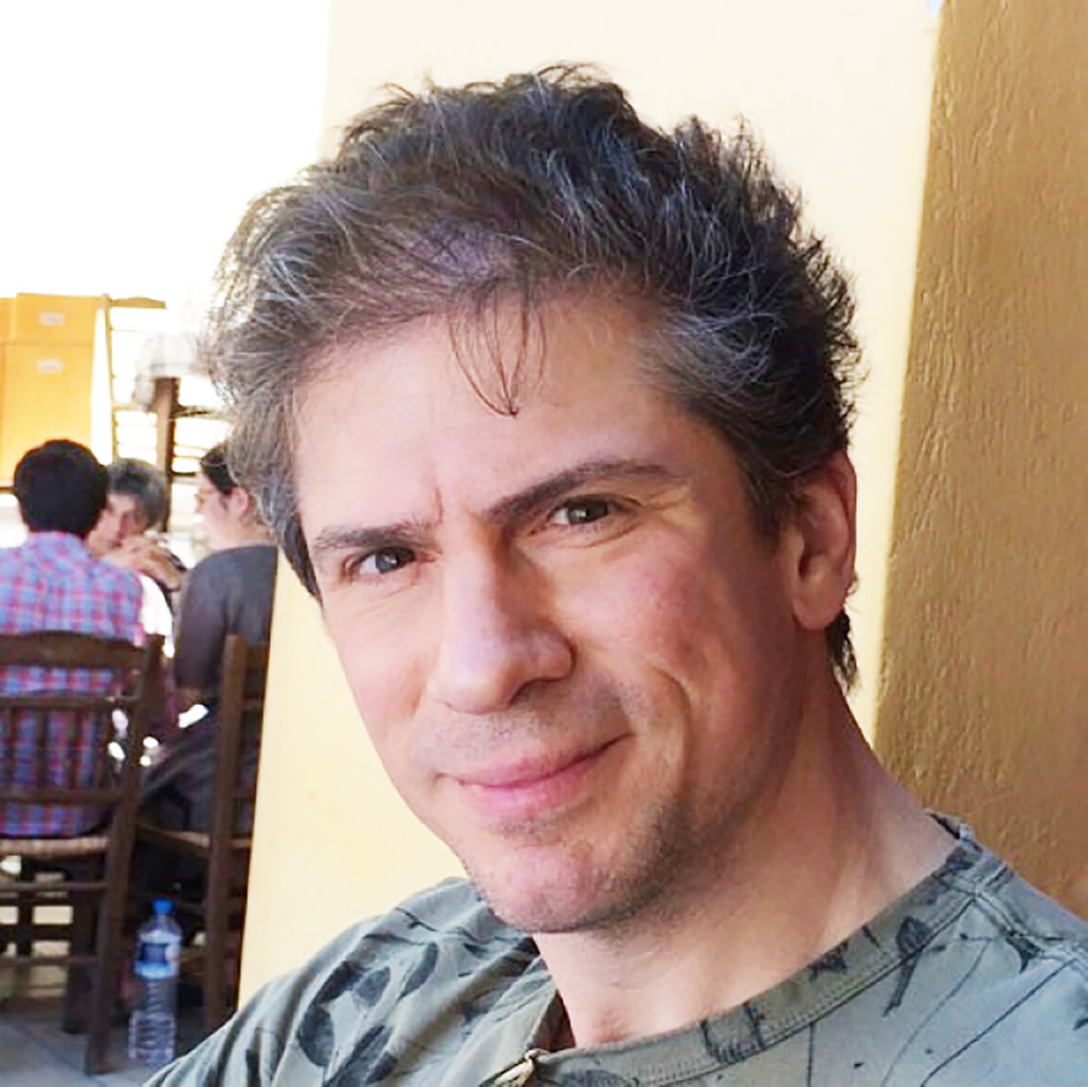
Dr. Serafim Batzoglou, Professor in the Department of Computer Science at Stanford University.

2016 marks the awarding of the inaugural ISCB Innovator Award, which honors an ISCB scientist who is within two decades of receiving a graduate degree, has consistently made outstanding contributions to the field, and continues to forge new directions. The inaugural winner is Dr. Serafim Batzoglou (Fig 1), Professor in the Department of Computer Science at Stanford University. Batzoglou will receive his award and deliver a keynote address at ISMB 2016 in Orlando, Florida, on July 12, 2016, to mark this honor.

Batzoglou remembers having an early fascination with numbers and a sense of wonder about the universe around him as a young child. He recalls, “I was interested in math and science for as long as I can remember. Before going to preschool, I remember counting and adding large numbers as well as wondering how big space is and where it ends.” Batzoglou’s curiosity was stoked by other cultural touchstones, including the novels of Jules Verne and Carl Sagan’s captivating *Cosmos* television series. These early experiences nurtured his interests in math, physics, and computer science, and Batzoglou went on to earn two bachelor of science degrees in mathematics and computer science at the Massachusetts Institute of Technology (MIT). Batzoglou also encountered computational biology for the first time as an undergraduate. He says, “Upon entering college, I was deciding between two fields of study that had fascinated me during high school: astrophysics and artificial intelligence (AI). However, during the early 90s, I felt that neither physics nor artificial intelligence was experiencing any exciting growth. At least, that was my impression around 1995, when I had been admitted to a PhD program in computer science at MIT and was deciding on an area to focus on. I took Bonnie Berger and David Gifford’s class on computational biology and felt that this was a research area with great potential where I could apply my computer science background to do science (rather than engineering).” Batzoglou credits his early mentors for giving him invaluable undergraduate research experiences, including Sorin Istrail, with whom he had a very enjoyable research summer during 1997, and his undergraduate research supervisor David McAllester.

Batzoglou went on to earn his PhD in computer science at MIT under the mentorship of his advisor Bonnie Berger and co-mentor Eric Lander. During his early career, he was the lead algorithms designer and implementer of ARACHNE, one of the first programs for whole genome shotgun sequence assembly that was used for assembling several genomes, including the mouse and dog genomes. Batzoglou’s early work included using comparative genomics for human gene identification. Together with his collaborators, he developed multiple genome alignment tools, including LAGAN, and applied these tools to predict human genes from similar mouse genes. This work was significant to the emergence of the field of comparative genomics and its applications for identifying genes, regulatory regions, and evolutionary events, as well as other features across species.

Batzoglou and his lab focus currently on the development of algorithms, machine learning methods, and systems for the analysis of genomic data. He recalls one of his more surprising research moments that stands out in his memory: “It was unexpectedly good news to me back in 2005 that Conditional Random Fields (CRFs) and similar flexible models that can learn very large parameter sets could be so successful in a large variety of classic computational genomics problems, including RNA secondary structure prediction, gene finding, and sequence alignment. My line of work on CRFs, together with my PhD students at the time, Chuong Do and Sam Gross, who led the effort, was when we began applying machine learning in earnest to genomics problems.”

Batzoglou aims to follow the example of his PhD mentor as he mentors students and postdocs in his lab: “I think I follow a similar style and philosophy as my PhD advisor, Bonnie Berger, in that I am supervising my students in a relatively hands-off, ‘first do no harm’ manner and, to the extent possible, allow them to define new research topics and directions.” He also looks forward to a time when more novel research can be supported through the grant system, saying: “The most talented and motivated students will do their best research when given freedom, encouragement, and some resources. I would be supportive of a large fraction, say 25%, of the government funds to be earmarked for work on new directions—i.e., work on which the proposing PIs [principal investigators] have no other funds and no related papers.”

Batzoglou’s novel research contributions have been recognized through several awards, including being named among the Top 100 Young Technology Innovators in 2003 by MIT’s Technology Review Magazine and a 2004 NSF CAREER Award. His research publications alone also show his impact on the field, and his purely bioinformatics-based publications have been cited hundreds of times. Batzoglou has also served the computational biology community in numerous capacities, especially through his service as a member of the steering committee and his service as a program chair, session chair, and organizing committee member for various RECOMB and ISMB meetings.

Looking forward, he says, “The topic I am most fascinated by right now, although it hasn’t majorly influenced my research yet, is deep learning. Like many of my AI colleagues, I subscribe to the opinion that we are witnessing a major breakthrough in our ability to replicate (and improve on) a large fraction of the intellectual and perceptual capacity of humans. The victory of AlphaGo against Lee Sedol is a historic moment. From a personal perspective, I learned Go in 2003, and back then I considered it a midpoint in AI between where we were and full-blown human-level intellectual capacity (excluding emotions and human experiences, which AI hasn’t been focusing on as much). The significance of advances in AI cannot be overstated. I believe that AI will transform medicine, finance, construction, manufacturing, commuting and transport, and almost every other sector in society over the next 20 years. I also believe that a large fraction of jobs in these fields will be made redundant. Re-education is great, but it is not clear at all what the new marketable human skills will be 20 years down the line. Perhaps anything involving human interaction, although that’s not clear. In terms of computational genomics and biomedicine, to the extent that we will be able to collect and agglomerate large genomic and biomedical datasets, application of AI will lead to breakthroughs that will start by vastly improving health care, agriculture, and biotechnology, and continue to places that are hard to imagine today.”

Batzoglou feels greatly honored to be selected as the inaugural winner of the ISCB Innovator Award, and says, “Innovation in computational biology—and in general—is largely a community process. I thank the committee for recognizing my work, and more importantly, I thank my colleagues, mentors, and foremost my students, with whom I should be sharing this Award.”

